# Self-management of a musculoskeletal condition for people from harder to reach groups: a qualitative patient interview study

**DOI:** 10.1080/09638288.2018.1485182

**Published:** 2018-10-28

**Authors:** Jo Adams, Wendy Lowe, Joanne Protheroe, Jill Lueddeke, Ray Armstrong, Cynthia Russell, Don Nutbeam, Claire Ballinger

**Affiliations:** a Faculty of Health Sciences, University of Southampton, Southampton, UK;; b Centre Medical Education Barts and The London School of Medicine and Dentistry Queen Mary, University of London, London, UK;; c Research Institute for Primary Care & Health Sciences, Keele University, Keele, UK;; d Richard Taunton’s Sixth Form College, Southampton, UK;; e University Hospital Southampton Foundation NHS Trust, Southampton, UK;; f Sydney School of Public Health, University of Sydney Australia, Sydney, Australia;; g Faculty of Medicine, University of Southampton, Southampton, UK

**Keywords:** Joint pain, arthritis, communication, accessible, experience

## Abstract

**Background:** This study recorded the functional health literacy levels of people with musculoskeletal (MSK) conditions from harder to reach groups and explored their experiences in engaging with health care professionals to self-manage their MSK condition.

**Methods:** We recruited participants, identified by key health and social care contacts as likely to have lower health literacy levels, and used semi-structured interviews to collect data. Thematic analysis was used to identify the main key themes arising from the transcribed interviews.

**Results:** Eighteen participants were identified and recruited from harder to reach community populations, 10 were scored as having inadequate functional health literacy on the Short Form Rapid Estimate of Adult Literacy Measure. Three themes were identified in relation to participants’ experiences of MSK self-management approaches: engaging with health care services; interpreting the health care providers’ message; and facilitating participation in MSK self-management.

**Conclusions:** Our findings indicate that people with a MSK condition, from harder to reach groups, experience multi-morbidity, find health care systems complicated and hear from health care professionals that their MSK condition cannot be cured. People interpreted that a lack of cure meant that nothing could be done to help their MSK pain. Engaging with self-management strategies was not seen as a priority for our participants. Strategies to simplify health communication, more time to process health information and supportive social networks helped our participants to understand and manage their MSK health on a day-to-day basis.

**Implications for Rehabilitation**
- MSK conditions are long term and prevalent in the UK with substantial impact on people’s daily life.
- Currently self-management strategies for MSK conditions are poorly communicated and many patients believe that nothing can be done to help their MSK pain.
- Good clinician communication that supports self-management is needed so that key messages can be effectively understood and used by patients with a range of literacy skills.
- Health services need to be even more accessible to help all individuals from a range of backgrounds better self-manage their MSK conditions.

## Introduction

The purpose of this paper is to present the background context and findings of a qualitative interview study. The study recruited people from patient groups living with a musculoskeletal (MSK) condition, who may have experienced barriers to accessing health care and defined as “hard to reach” [1] by health researchers. We explored their experiences of accessing and engaging with self-management strategies.

Globally, MSK conditions are the second largest cause of disability [2]. The personal impact of living with a MSK condition can be formidable and associated with high costs to individuals and health services. Effective self-management interventions can help to reduce these costs and are identified as a priority implementation area for health services [3].

Self-management strategies for people with long-term conditions are complex interventions that include patient education and behavior modification [4]. They are designed to encourage people to take an active self-management role to improve health outcomes [5]. For people with MSK conditions, self-management interventions may include specific targeted regional exercises, general exercise, activity modification and pacing, joint protection techniques, and use of orthoses [6].

Generic, non-disease specific, formal self-management programs have been developed and tested with varying success. Among the more effective programs are the Expert Patient Programmes [7] and the Arthritis Self-Management Programme [8]. Some individual intervention components of these generic programs have evidence of effectiveness for a MSK population including, for example, resistance exercises [9]; individual counseling and cognitive behavioral programs [10,11]. However, the benefits of self-management interventions have not been universal. Many self-management interventions have failed to accommodate the varied needs and skills of more disadvantaged populations, particularly those populations from harder to reach groups with lower health literacy levels [12].

“Health literacy” describes the cognitive and social skills that determine the motivation and ability of individuals to gain access to, understand and use information in ways which promote and maintain good health [13]. Nutbeam’s model [14] identifies three levels of health literacy; functional interactive and critical. Functional health literacy includes the reading and writing skills that enable someone to access and navigate health care information and settings. Interactive health literacy includes skills that support people to identify meaningful information from a variety of communication methods and apply this to their own situation. Critical health literacy includes skills that enable people to analyze and apply information to exert greater control over their own lives. Health literacy is related to but independent from general literacy and numeracy skills. Health literacy is context specific. The organization of the healthcare system can make it easier or harder for people with lower health literacy to successfully navigate its demands [15]. Social resources (social networks, family support) as well as personal skills (literacy and numeracy) are important in using health information and services [16]. Health literacy is important as individuals with lower health literacy are more likely to have lower activation to engage with personal self-care [17], increased difficulty following medical advice, adopting self-managing strategies and worse functional outcomes [18–22]. For health care approaches that require active engagement of patients, such as self-management, this is particularly pertinent [23]. It is already known that the impacts of MSK patient self-management programs are not equivalent for people with different levels of literacy [24] and suggests that health literacy is important when exploring the impact of self-management approaches for patient populations.

Self-management education is recommended as a core intervention for people with MSK conditions [25,26]. It is understood that people with higher levels of health literacy gain greater knowledge, faster development of skills, and increased confidence to engage in effective self-management practices than people with lower levels of health literacy [20,22]. However, there is little evidence relating to how people with lower health literacy levels engage with self-management programs, and further research is warranted [27].

The overall aim of our study was to access and recruit harder to reach populations with suspected lower health literacy levels living with a MSK condition and explore their experiences and understandings in managing their MSK condition. Specific objectives included:

- To explore how people with a MSK condition understand and manage their condition.
- To explore peoples’ experience of MSK supported self-management approaches and any difficulties they may encounter.
- To explore strategies to facilitate supported self-management for people when dealing with healthcare services and professionals.

## Design and methods

We combined the use of objective, standardized measures of functional health literacy with an exploration of the subjective experience of patients living with and managing their MSK condition. Informed by pragmatism, the use of quantitative and qualitative approaches is acknowledged to be particularly relevant in understanding the management of multiple chronic conditions in context [28,29].

Our procedures integrated public health ethical principles for researching harder to reach community populations [30]. In line with UK best research practice guidelines [31], study materials and our pilot interview schedule were developed with the assistance of a Patient and Public Involvement (PPI) representative who self-identified as having a lower level of literacy, and a long-term condition and volunteered to join the university’s PPI network. The study researcher (WL) was experienced in working alongside marginalized populations. Interviews were chosen as characterized by a fluid, informal structure, they permit in depth exploration of perspectives concerning experiences and knowledge [32] and enable the researcher to orientate to the individual participant’s world-view [33].

### Sample and recruitment

People aged 18 years and over with a chronic, physician diagnosed, MSK condition were eligible to take part. People were excluded if they had cognitive or neurological impairments or a learning disability, spoke English as a second language or had a visual impairment. Key community health, social care, or educational contacts from secondary care rheumatology clinics, primary care General Practitioner (GP) surgeries and community groups across southern, central, and northern England, identified potential participants as having a lower level of health literacy through their own prior knowledge of the individual. The key community contacts introduced the study at the end of a routine appointment and the research fellow contacted interested potential participants. Inclusion criteria were checked and an appointment made for a home interview with participants.

### Data collection tools and procedure

At the interview, the researcher and participant reviewed the written Participant Information Sheet orally and obtained written consent. An interview schedule guided the interview (Table 1). All interviews were digitally recorded and transcribed verbatim.

**Table 1.** Semi-structured interview schedule.

**Introduction:** Explanation of project, consent forms, expenses, demographic data, confidentiality
**Understanding and management of MSK condition**
1. Can you tell me about your arthritis? (Prompts: how did it develop, how does it impact on your life, what do you find helpful in managing it?)
2. How does having difficulty reading, writing or doing maths affect you doing this?
(Prompt: what barriers do you have to managing your arthritis? What helps you?)
**Exploring participation in MSK patient education**
3. Have you ever taken part in patient education for your condition?
(Prompts: When was this? What was it about? What was it like?)
4. Did the patient education help you to manage your condition on a day to day basis?
5. Did the patient education help you in overcoming any difficulties? How?
6. In your experience, what information was most helpful in helping you manage?
7. In your experience, what information was least helpful in helping you manage?
**Strategies to overcome difficulties when dealing with health professionals**
8. What do you do to help overcome trouble with reading, writing and maths?
(Prompt: do you have any trouble working out when and how much to take of your tablets, completing forms, dealing with hospital appointment letters, for example? What helps in these situations?)
9. What do you think you need **to know** to effectively deal with your condition?
10. What do you think you need **to be able to do** to effectively deal with your condition?
11. Can you think of some different ways in which health professionals might be able to give you information that would help you deal with your condition better?
12. What do you think health professionals need to know to help you manage your condition?
13. What do you think health professionals need to be able to do to help you manage your condition?
14. How do you decide what works for you and what doesn’t? What would make a difference to managing your arthritis for you?
**Exploration of broader health literacy issues**
15. What are your thoughts on hospital sites measuring literacy levels of patients so that they would know if people needed extra help?
16. Could you tell us your thoughts on patients being identified at hospital by a sticker on their notes or something that tells the staff that the person has lower levels of literacy?
17. Can you think of anything else that might be useful to know when thinking about patient education and MSK conditions?
18. What would you say are your priorities in life?
(Prompt: if you had to list them in order of importance, what would the first one be? Where does your health and arthritis fit in?)
**Summary** Any other points, thanks, summary. Completion of REALM-SF. Finish.

The Rapid Estimate of Adult Literacy Measure Short Form (REALM-SF) [34] was used post-interview to record a standardized measure of functional health literacy. The REALM-SF has accepted levels of reliability and validity and is widely used, however, it does not measure interactive or critical health literacy [35]. We chose not to start with the REALM-SF to avoid a “test” like start to the interview. Participants also verbally completed a PROGRESS-Plus questionnaire [36] to provide background demographic information.

### Data analysis

All transcribed interviews were analyzed thematically, using Braun and Clarke’s staged approach [37]. The process of data analysis involved continual reflective cycling between interpretation and experiential annotation of data. Points of divergence, convergence, commonality and nuance were noted. Iterations of the emerging thematic framework were carried out by two members of the research team (JA, CB) to promote trustworthiness [38]. As the analysis progressed these two members reviewed, checked, and agreed the emerging themes. Where there was disagreement this was resolved through discussion. Additionally, reflective memos were made as analysis proceeded, to capture the interpretative process, and keep a record of developments in thinking. Illustrative excerpts from the data are provided to illuminate the themes and offer context-specific examples to support the validity of the interpretation [39].

### Ethics

Full University of Southampton and NHS Ethics approvals were obtained. All data were stored securely on password-protected university computers. Participants were referred to by participant number and study pseudonym and all identifying details were anonymized in transcripts, and iterations of findings.

## Results

### Participant characteristics

Eighteen participants (nine female; nine male) with an average age of 53.3 ± 28.6 years took part. Participants had an average of 3.2 comorbidities. Four participants reported having no other comorbidities alongside their MSK condition. All other participants reported a combination of other non-MSK comorbidities including; cardiovascular, pulmonary, renal, gastrointestinal, ophthalmic, skin, and mental health disorders. A third of the participants reported having depression. The participants were predominantly white British, and most had completed high school (Table 2). All participants accessed healthcare through their primary care GP surgery whilst three quarters accessed secondary care. Table 3 shows recruitment per location.

**Table 2.** Participant’s demographic data.

Participant	Ethnicity	Age	Occupation	Gender	Education	Socio Economic Situation (Income: £per calendar month)	Social Support	Comorbidities	Disease Diagnosis	Physician diagnosed	Disease duration (Years)	NHS Attendance	REALM-SF (Range 0–7)
Bill P1	White British	66	Disability Allowance	Male	College	£1001–£1500 pcm	Married, social network, community ties	Chronic kidney disease, Chronic obstructive pulmonary disorder, Osteoporosis, Heart Failure	Hip Arthritis	✓	2	Primary care Secondary Care	7
Viv P2	White British	64	Disability Allowance	Female	No data	£0–£500 pcm	Lives alone, sister & brother, neighbor, volunteers	Asthma,	Hip Arthritis	✓		Primary care Secondary Care	7
Jane P3	White British	43	Disability Allowance	Female	High School	£0–£500 pcm	Lives alone, family, too much effort to be involved socially or community	Anxiety, depression	Fibromyalgia	✓	5 – 10 years	Primary care Secondary Care	7
John P4	White British	70	Retired	Male	High School	£501–£1000 pcm	Married, two daughters, grandchildren, strong community & neighborhood, workers like family	Pleural plaque, glaucoma,	Arthritis	✓	>20 years	Primary care Secondary Care	7
Linda P5	White British	46	Disability Allowance	Female	College	£0–£500 pcm	Family, friends	Post-traumatic depression	Fibromyalgia, Chronic low back pain	✓	2 years	Primary care Secondary Care	5
Joyce P6	No data	61	Disability Allowance	Female	7 – 11^th^ Grade	£0–£500 pcm	Family	Depression, chest infection	Chronic MSK pain	✓	15 – 20 years	PC	0
Rita P7	White British	47	Disability Allowance	Female	7 – 11^th^ Grade	£0–£500 pcm	Family, talks with neighbors	Asthma, breathlessness, depression	Arthritis, chronic pain	✓	10 – 12 years	PC	0
Brian P8	White British	60	Disability Allowance	Male	7 – 11^th^ Grade	£0–£500 pcm	Family, talks with neighbors	Emphysema,	Gout	✓	3 years	Primary care Secondary Care	1
Frank P9	White British	82	Retired	Male	High School	£0–£500 pcm	Strong family support, wife & son		Rheumatoid Arthritis	✓	54 years	Primary care Secondary Care	7
Anne P10	White British	68	Retired	Female	High School	£0–£500 pcm	Strong family support, husband, siblings	Asthma, diabetes, Chronic Kidney disease, Hypertension	Osteoarthritis	✓	10 years	Primary care Secondary Care	7
Tim P11	White Other	30	Employed	Male	No data	£501–£1000 pcm	Strong family		Osteoarthritis	✓	No data	Primary care Secondary Care	6
Adrian P12	White Other	29	Employed	Male	No data	£501–£1000 pcm	Close community		Rheumatoid Arthritis	✓	20 years	PC	6
Carole P13	White Other	31	Employed	Female	College	£0–£500 pcm	Lives alone, looks after herself	Asthma, Bipolar disorder	Knee Patellofemoral pain	✓	5 years	Primary care Secondary Care	6
Rachel P14	White British	50	Disability Allowance	Female	High School	£0–£500 pcm	Very close family, strong community & neighborhood	Psoriasis, Depression	Rheumatoid Arthritis, Generalised Arthritis Low Back Pain	✓	24 – 30 years	Primary care Secondary Care	0
Kate P15	White British	46	Employed	Female	High School	£0–£500 pcm	Strong family, strong neighborhood		Arthritis	✓	2 years	PC	7
Bob P16	White British	39	Disability Allowance	Male	High School	£0–£500 pcm	Brother, dangerous neighborhood	Asthma, Hiatus Hernia, Creutzfeld Jacob syndrome, Migraine	Arthritis	✓	18 years	Primary care Secondary Care Community Hospital	4
David P17	White British	66	Retired	Male	High School	£0–£500 pcm	Strong family, neighbor	Multiple strokes, melanoma, Low blood pressure	Gout	✓	2 years	Primary care Secondary Care	4
Chris P18	White British	62	Employed	Male	College	£1001–£1500 pcm	Wife, son, extended family	Mild Hypertension, Enlarged Prostate, Anemic, Cataract, Elevated cholesterol	Osteoarthritis, rheumatoid arthritis	✓	No data	Primary care Secondary Care Community Services	7
Total *n* = 18													Lower level of literacy <6 n = 10

**Table 3.** Recruitment across the UK.

	Number of participants per location								
Setting	South	Literacy ≤6*	Literacy >6*	Central	Literacy ≤6*	Literacy >6*	North	Literacy ≤6*	Literacy >6*
Primary Care	5	1	4	3	3	o	2	1	1
Secondary Care	3	1	2	N/A			N/A		
Community groups; FE Colleges	5	4	1	N/A			N/A		

* Literacy ≤6 indicates a low functional health literacy score; Literacy >6 indicates an adequate functional health literacy score as measured by the REALM- SF.

Across locations and settings, recruitment from central primary care and south community showed the largest proportion of identified participants as having lower levels of functional health literacy. Nine participants were on Disability Allowance, four were retired and five were employed.

### Identifying people with low health literacy

Key contacts identified individuals for this study who were thought to have lower levels of health literacy. However, eight participants scored as having adequate functional health literacy when assessed using the REALM-SF (i.e., score >6). All participants’ data regardless of their functional health literacy level was included for analysis. The themes identified did not differ between those participants with higher or lower functional health literacy levels as assessed by the REALM-SF.

### Themes

Three themes were identified:

- Engaging with healthcare services
- Interpreting the healthcare provider’s message
- Facilitating participation in MSK self-management

### Theme 1: Engaging with health care services

This theme covers participants’ experiences of trying to navigate access to health care advice in clinical settings and the strategies people used when need to do so. It covers accessing NHS health services in England and the process of dealing with face-to-face personal communication. The complexity of engaging successfully with the health care system and ways that facilitated engagement are presented below.

### Accessing health services

Participants reported that they developed an understanding of their MSK condition via a variety of routes – through written health care information, internet searches, formal medical diagnoses, investigative tests, prescribed medication, and treatment. This included health professional advice and patient education provided during individual consultations. Rachel appreciated how her doctor would take time to explain medical terminology:

You know she did explain that. She does explain everything to me. (Rachel – P14, REALM 0, Low Functional Health Literacy).

Whilst Bob was able to study and make use of written health care leaflets:

Like when I’m in hospital and the anaesthetist gives me some kind of form, I take them home I tend to look at them and take them home. (Bob – P16 REALM 4, Low Functional Health Literacy).

Others accessed online resources and sought help from friends and/or relatives to help access health information. Joyce relayed how she relied on her social networks to help her understand written health information:

Some letter came and you know I have to wait for somebody to read for me. Sometimes you know I was sitting and I’m trying to read and I can’t read. And then I’ll look for somebody and, ‘Can you read this for me please. ’ (Joyce – P6, REALM 0, Low Functional Health Literacy).

Whilst Tim outlined how he would access additional online web based health support from other charitable networks:

But then we have a lot of charities which you can call or you can find online, support for all this. (Tim – P11, REALM 6, Low Functional Health Literacy).

Participants expressed a wide range of responses to the challenge of engaging with their health care services to manage their MSK condition. At one extreme, Brian talked about physically “hiding” in order to avoid engaging with a complex health care processes with which he felt overwhelmed:

Having this (i.e., low health literacy), seriously, having this. I was hiding, yeah, I was hiding, definitely hiding. I won’t, you know, I wouldn’t go to these places if I know I gotta read and sign and write things. (Brian – P8, REALM 1, Low Functional Health Literacy).

However, other participants appeared to be at ease with being open and sharing their own difficulties in accessing health care services. They understood that it was important to have their health information explained to them in clear, uncomplicated terms. Rachel, for example, readily disclosed that she has difficulties understanding and reading. She seemed comfortable that her lower levels of literacy were noted in her medical records and identified that this was a helpful approach for her:

Because it’s on me notes, yeah (i.e., challenges with health literacy) … Sometimes, … they change these big names, you know and that …. Well she’s just put it in a way for me: “It’s painful joints”, you know, ‘It’s all your joints’, because she knows I’m no good at reading. (Rachel -P14, REALM 0, Low Functional Health Literacy).

### Complexity of communication

In comparison, some patients identified that the health information they were given was too complicated and rhetorically located the “problem” with the health service providers. Viv, reflects:

I don’t think it’d matter how educated or if you could read or write. It’s just that when you’re not a doctor that you can’t understand their words, their long words and their explanations. So I think sometimes, that’s why they need to come down to our level, not that we’re thick or anything, or stupid. (Viv -P2, REALM 7, Adequate Functional Health Literacy).

In this excerpt, Viv, who has adequate health literacy levels, suggests that the challenge lies with the doctors and their “long words”, rather than her own lack of ability. She identifies that the medical jargon used is too complex, inaccessible and inappropriate for her. She effectively refutes any negative attribution, such as being “thick” or “stupid” and clearly identifies the health care professional’s communication skills as lacking. Whilst she implicitly identifies a hierarchy where the patient is at a lower level than the doctor, she questions the ability of the health care professional to communicate clearly what she needs to know. She is confident that the language used is too complex for lay understanding and inappropriate for patients.

We detected no evidence from our participants that the services or clinical pathway they would have been part of was clear, organized or even apparent for them. The clinical communication and consultation tended to be described as time-limited, rushed and too short. There were limited examples of participants being encouraged to engage in self-management activity, beyond being provided with a patient information leaflet. John, with adequate health literacy, describes receiving what he perceives as a chaotic, time pressed health service that leaves him feeling bewildered:

Yeah, it’s just that you go to these hospitals and you could be sat there for an hour or two waiting because they’re behind, they’re obviously busy and then you just, as I said, you just go through these big words they say to you and goodbye. There’s nothing…. I don’t even know sometimes what I've ended up going there for. Because I haven’t got a clue when I come back. (John – P4, REALM 7, Adequate Functional Health Literacy).

### Theme 2: Interpreting the health care providers’ message

This theme includes participants’ understanding of the health care messages received from face to face contact with health care professionals. For many we interpreted that the communication with health care professionals about the MSK condition influenced how people understood the condition and what role they understood they could contribute to managing their MSK pain. Mostly, participants reported this to be limited. Participants did not offer positive or hopeful examples of engaging with self-management from their health care professionals. For example, Brian recalls:

Well the only information you get is what they you know if it’s, if I got gout all I can do is take a tablet. The thing is what else can they do? I don’t see, I don’t see any talk, no disrespect to you, I don’t see any talkin … I don’t know. All they turn around and said is ‘go home and rest it’ That’s all they could say. (Brian – P8, REALM 1, Low Functional Health Literacy).

We interpreted that self-management was therefore not seen as a priority for participants as they had not heard about potential strategies from their health care providers.

### Missed opportunities at diagnosis

Many participants regardless of health literacy level recounted how when they had been told about their MSK diagnosis they heard at the same time that there was little that could be done to help. The message participants received was often confusing, as illustrated by Bill:

You got booklets out anyway explaining it all, but it’s not … it does never seem plain enough to me for people to understand it properly you know, they come out with all these ‘osteoarthritis’ and, but they don’t … they just tell you, you’ve got it, they don’t explain exactly what it is. (Bill – P1, REALM 7, Adequate Functional Health Literacy).

Whereas, Anne recounts her experience of being diagnosed with osteoarthritis recalling that the approach discussed was medication management, even when actively asking what “we” can do:

the x rays’ he said ‘it’s called osteoarthritis’ I said Yeah…… so? He said ‘What do you mean so?’ Well what are you going to do about it? He said ‘Well we might be able to get it under control, but you might not, you might have to live with it’. So I said Well… …. Will I end up in a wheelchair? He said ‘in all probability, the answer is yes’ … I said what can we do? He said ‘we can keep it under control with drugs. (Anne – P10, REALM 7, Adequate Functional Health Literacy).

### Pharmacological pain management

There seemed to be a general pessimism from participants about managing their MSK pain on a daily basis. As participants heard that nothing could be done to cure their MSK condition, they interpreted that nothing could be done to help or alleviate their MSK joint pain. Rita was pessimistic and believed that nothing could be done to help:

There’s not much that you can, that the doctor can do for arthritis really. (Rita – P7, REALM 0, Low Health Literacy)

Rachel reported a similarly negative, rather hopeless outlook:

I’ve been to … I’ve had appointments and that but there’s nothing. As I’ve said there’s nothing that … they said to me that there’s nothing they can do for it. (Rachel – P14, REALM 0, Low Functional Health Literacy)

The potential for optimizing a self-management approach seemed to have been missed on many occasions:

Well the only information you get is what they know….all I can do is take a tablet. The thing is what else can they do? (Brian – P8, REALM 1, Low Functional Health Literacy)

David reflected that the written patient education leaflet he was given would not have any impact on how he lived his life from day to day anyway:

…but I mean, leaflets are not going to do much good about it. I live my life as I live my life. I don’t think a leaflet will change that. (David – P17, REALM 4, Low Functional Health Literacy)

Beyond taking medication, there were no accounts of other self-management strategies included in our participants’ accounts:

Even then they said, ‘Rachel, you’re on the strongest tablets. Go away!’ (Rachel – P14, REALM 0, Low Functional Health Literacy)

Participants appeared to have a general pessimism about the medical management of their MSK pain which then appeared to leave no opportunity for raising expectations about alternative supportive self-management approaches. When health professional support and advice for their MSK pain was accessed our participants recalled receiving only advice on medication management. Self-management strategies were mostly absent in participants’ accounts and there were no examples offered of any non-pharmacological approaches to managing their MSK pain. People did not express hope in being able to successfully managing their condition and no participant recounted or described a clear partnership model of care, whether this was with a GP, rheumatologist or any other health professional.

### Theme 3: Facilitating participation in MSK self-management

This theme presents strategies identified and reported as supportive by people with both low and adequate health literacy levels living with their MSK condition. The theme is informed by participants recalling few instances of receiving support for self-management from health care professionals. More supportive examples were identified from participants’ local and community networks.

### Importance of personal contact and local networks

The importance of face-to-face contact with local health care professionals was valued. Participants appreciated practical support from those health care professionals, in a position to provide this. The excerpt below, for example, includes reference to a pharmacist who dispensed medication organized in a tablet dispenser box to help support the patient on a regular basis.

…. I’ve had appointments and that but there’s nothing. …….… So I’m just wasting their time and like, in a way, my own. ‘Cos I’ve got all my tablets, they have them done every week. (Rachel – P14, REALM 0, Low Functional Health Literacy)

Tim identified his community general practitioner as the preferred point of contact for health care information and support:

We would have liked to have had it (health care information) from the GP. (Tim – P11, REALM 6, Low Functional Health Literacy)

Participants approached friends, family and social networks for personal and ongoing support. Carol identifies her reluctance to seek medical or health support when in pain:

When I’m in pain I don’t tend to want to go the hospital I won’t even go to the GP and I’ll just go and find out for myself …. And that’s when sort of, it would be a last resort to go to the doctor. (Carole – P13, REALM 6, Adequate Functional Health Literacy).

David recounts how he sought support from his neighbor in helping him manage his MSK health care needs:

I’m dyslexic so I don’t read very well … If I have a problem, you know, my neighbour helps me. But you know what I mean, I get by, do you know what I mean? (David – P17, REALM 4, Low Functional Health Literacy)

### Practical personal strategies

There was evidence of positive strategies and approaches used by participants to facilitate the engagement with and understanding of health care information provided by health care professionals. Participants described “going over the words” when given written patient information, and when time permitted, requesting clarification from health providers. Participants frequently spent more time studying such information at home, seeking support from a range of additional resources, including charities, voluntary organizations, friends, family and neighbors to help understand the information provided by the health care professional. Written information, although often referred to as complicated, was useful for participants to take home and read again.

## Discussion and conclusions

### Discussion

Our research identified that people with lower and adequate levels of functional health literacy who were managing a MSK condition had difficulties in engaging with health care information. Participants heard from health care professionals that their MSK condition and pain could only be managed by taking medication and that their MSK condition could not be cured. Discussions about the role that self-management approaches could contribute to their overall MSK management were largely absent from our participants’ health care consultations. This left people feeling hopeless about managing their MSK condition and pain. However, participants did give examples of practical strategies and support from local personal networks that helped them navigate health care information and contribute to managing their MSK condition on a day-to-day basis.

Our findings build upon previous research that indicates that patients with lower levels of health literacy are less likely to engage in prevention activities and self-management approaches [40,41]. We also provide further evidence that MSK self-management support is often not provided in line with national guidelines [42]. We found that health care advice was limited to advice about medication, and provision of written leaflets. Our findings reinforce others’ who have already reported the mismatch between the complexity of health information and patients’ ability to understand this information [22]. The health care information received by our participants did not support them in adopting self-management behaviors. It appears that patient education materials in use did not actively build patient knowledge, understanding, and confidence to act on information that is known to support people engaging with self-management approaches [43,44]. Here, lessons could be learned from commercial, for-profit, MSK websites that present information in clear, more patient friendly and persuasive ways than many other not for profit charitable and National Health Service sites in the UK [45].

Our participants offered examples of good practice where health care staff demonstrated effective communication skills but also examples where this was not the case. It was evident from our findings that interactions with health care professionals need to go beyond information giving so that understandings of patients’ disease perceptions can be understood and checked. By way of example, the use of “teach back” strategies is known to improve understanding in health care scenarios [46] and could be a useful routine addition for consultations. Participants reported examples of sensitive individualized approaches. These were supported by the identification of any literacy details on medical notes and health care professionals who were able to tailor their consultations with time allowed for further explanations and questions.

Participants also observed that where personal, face-to-face communication was compromised this was further compounded by health care environments that were seen to be too hectic, busy, and confusing for effective communication. These findings have also been previously reported in other health care settings [47] and further illustrate the importance of creating a more supportive environment for effective communication than is common in most clinical settings at present.

Our participants reported that the experience of living with and managing a MSK condition was mitigated through relationships with others outside of hospital and clinical environments. Family, friends, and neighbors were important to our participants in supporting them to live with and manage their MSK pain. This resonates with other recent research that indicates that community networks and people’s existing personal network provides the basis for greater connectivity and engagement with self-management support opportunities [48]. Contacts with health care professionals and clinical environments are fleeting for patients, further reinforcing the need for health care information and advice to fit with individuals’ everyday life and their personal circumstances. The value of considering the contextualized personal and social issues of people living with joint pain has already been identified as important in supporting effective self-management of MSK joint pain [49]. Our findings also suggest that it is timely to consider more creatively how self-management support can be provided outside of traditional clinical settings. Engagement with community housing association partners, charitable physical activity clubs associated with national sports teams and city library services serve as potential viable options for long-term condition management [50].

We chose to use the REALM-SF to measure levels of functional health literacy in this research, as the tool is widely used, practical, reliable, and valid in our sample population [34]. However, this is a uni-dimensional measure of health literacy and cannot report higher levels of health literacy (interactive and critical) that are required for improved and sustainable health care self-management outcomes. Ten of the 18 study participants had lower functional health literacy according to the REALM-SF. These results suggest that measuring functional health literacy alone is insufficient to identify the skills and support people need to successfully navigate and interact with health care systems and professionals and evaluate self-management options. The limitations of the REALM-SF have been noted previously [51,52]. Our findings also reveal some dissonance between health literacy as measured by the REALM-SF and the subjective assessment of the health care professional. This suggests that identifying people with differential levels of functional health literacy is difficult for health care professionals. Over 40% of our sample, with adequate functional health literacy, recalled similar experiences to people with low levels of functional health literacy. We detected no difference in the strategies identified for self-management by participants with adequate or low levels of functional health literacy. This suggests that interactive and critical health literacy play crucial roles in the skills required by people to engage with self-management in MSK conditions. It also indicates that a universal approach to supporting accessible health care environments and processes can enable people with different levels of functional health literacy to better self-manage their MSK health care needs. For future studies, inclusion of a health literacy assessment tool, such as the Health Literacy Questionnaire [53] that has been designed alongside patients and clinicians and measures all dimensions of health literacy is advised. This is more likely to capture the skills and support necessary to manage health care needs.

Whilst we believe we were successful in accessing and recruiting people from groups who often are not traditionally recruited into research, this approach can be further improved to continue to widen the support and appeal of participating in research projects for people with lower levels of health literacy. The involvement of a patient representative to help review written paper information was useful. We also believe a flexible approach to home interviewing and less formal language for participant information sheets was helpful in accessing and engaging this population.

Care has to be taken not to over-interpret the results from this study but the findings from our study are consistent with similar studies examining the quality of communication and its effectiveness with people with lower literacy levels with a range of chronic conditions and in different organizational settings [40,54,55].

### Conclusions

In order to support and facilitate self-management approaches for people with MSK conditions, a reconsideration of how supported self-management for people with MSK conditions is delivered and communicated by health care practitioners is required. The preparation and continuing education of health care professionals to gain the skills necessary to communicate complex health care information requires ongoing attention and focus. This would better equip health care professionals to understand the impact of low health literacy on patients’ ability to understand and respond effectively to written and oral communication. This will help to ensure that key messages can be effectively understood and used by patients with a range of health literacy abilities to support the self-management of MSK conditions.
